# The Moderating Effect of Mindfulness on the Mediated Relation Between Critical Thinking and Psychological Distress via Cognitive Distortions Among Adolescents

**DOI:** 10.3389/fpsyg.2019.01455

**Published:** 2019-06-26

**Authors:** Michael Ronald Su, Kathy Kar-man Shum

**Affiliations:** Department of Psychology, The University of Hong Kong, Pokfulam, Hong Kong

**Keywords:** mindfulness, critical thinking, cognitive distortions, psychological distress, moderated mediation

## Abstract

Critical thinking has been widely regarded as an indispensable cognitive skill in the 21st century. However, its associations with the affective aspects of psychological functioning are not well understood. This study explored the interrelations between trait mindfulness, critical thinking, cognitive distortions, and psychological distress using a moderated mediation model. The sample comprised 287 senior secondary school students (57% male and 43% female) aged 14–19 from a local secondary school in Hong Kong. The results revealed that high critical thinking was significantly associated with high levels of psychological distress when mindful awareness was low among adolescents. Trait mindfulness was found to moderate the indirect effects of critical thinking on psychological distress via cognitive distortions as the mediator. Specifically, in low trait mindfulness conditions, critical thinking was found to associate positively with cognitive distortions and psychological distress. Such associations were not observed in high trait mindfulness conditions. The findings reveal that though critical thinking has positive associations with cognitive functioning, its associations with affective well-being might be negative. The results also suggest that mindfulness might play an important role in preventing the possible psychological distress associated with critical thinking. Educational implications relating to the fostering of critical thinking and mindful awareness are discussed.

## Introduction

### Critical Thinking as a 21st Century Skill

With the fast-paced technological advances, the rise of the “knowledge economy,” and the information overload brought about by the internet, educators from all over the world have been re-evaluating the skill sets that are vital for people to thrive in the 21st century. Critical thinking is one of the most frequently reported and cited 21st century skills (Ananiadou and Claro, 2009;Dede, 2010). Research has shown that critical thinking is associated with numerous positive cognitive outcomes such as academic attainment (Bitner, 1991), cognitive performance (Witt, 2002), decision-making abilities (Shin, 1998), and decreased cognitive bias and heuristic thinking (Facione and Facione, 2001). It has been found to be a better predictor of life decisions than intelligence (Butler et al., 2017). The benefits of critical thinking are particularly prominent in the 21st century when the internet has become the essential vehicle of information. Anyone can now create “information” online. It is critical thinking that allows people to judge the truthfulness, credibility, and bias of information they come across in online environments (Leu et al., 2014) and synthesize the information into a coherent knowledge base (Brand-Gruwel and Stadtler, 2011). In addition, as scientific research and findings are becoming more accessible to the general public, a certain degree of scientific literacy is highly desirable, if not essential. Such scientific literacy can be developed by learning the skills and techniques in critical thinking (Britt et al., 2014). Critical thinking has also been shown to be useful in social and interpersonal problem solving and decision making (Ku, 2009).

The benefits of critical thinking are well researched and established in the cognitive and, more recently, the social domains. However, less is known about the relation between critical thinking and the affective aspects of psychological functioning. The goal of our study is to explore such possible relations. As critical thinking skills have been endorsed and promoted by various education systems around the world (Australian Council for Educational Research, 2002; Association of American Colleges and Universities, 2005), the findings of our study may have important implications for the educational practices across the globe regarding the development of critical thinking skills among students, especially adolescents. This is because the development of higher order cognitive processes is believed to be most active in late adolescence and early adulthood (Sowell et al., 1999). The development of critical thinking is found to be less mature in children (Giedd et al., 1999) and many critical thinking inventories have suggested minimum age for administration (Halpern, 2003; Ennis et al., 2005).

Critical thinking is broadly defined as “reasonable reflective thinking that is focused on deciding what to believe and do” (Ennis, 1987, p.10). Halpern (2003) provides a more elaborate definition by referring to it as “purposeful, reasoned, and goal-directed… thinking involved in solving problems, formulating inference, calculating likelihoods, and making decisions (p.6).” In this sense, the word critical is not meant to imply “criticism” or “fault-finding” but careful evaluation or judgment that aims at improving the thinking process (Halpern, 1998). Critical thinking is considered to be a trait instead of a state. A trait is thought to be a personal characteristic that remains comparatively stable across time and situations, whereas a state is considered to reflect one’s adaptation to a specific circumstance (Hamaker et al., 2007). For the current study, the working definition of critical thinking is based on the conceptualization of Ennis (1987) which has a narrower focus on one’s ability to make inferences based on (1) induction, (2) credibility of sources and observation, (3) deduction, and (4) assumption identification.

### Possible Relations Between Critical Thinking and Affective Functioning

Very few research studies have explored the relation between critical thinking and its effect on affective functioning. Among these studies, Scott (1983) empirically explored the relation between critical thinking performance and state anxiety level in 85 women (aged 18–60) prior to experiencing breast biopsy and 6 to 8 weeks post-procedure. Only participants later found to have benign conditions were included and post-tested. The results showed that state anxiety and critical thinking ability were not significantly correlated pre- or 6–8 weeks post- biopsy. Scott did notice a negative relation between critical thinking performance and state anxiety in participants from the high-anxiety subgroup. It was hypothesized that after reaching a certain level of anxiety, critical thinking ability begins to decline as a function of high anxiety. However, no proper moderation analysis with state anxiety as the moderator was conducted. Also, the criterion for classifying participants into the high anxiety subgroup was not provided. It appears questionable to analyze the relationship of a variable after dissecting it into extreme groups without sound justifications.

Irwin and Bassham (2003) theorize that critical thinking might influence affective functioning based on the theory of cognitive therapy. Cognitive therapy maintains that dysfunctional or maladaptive thinking is the root cause of psychological distress (Beck, 1976; Beck and Weishaar, 2000). Dysfunctional thinking is characterized by the presence of systematic errors in reasoning, also known as cognitive distortions, which in turn compromises people’s mood and behaviors. Common cognitive distortions include arbitrary inference, false dichotomy, selective abstraction, overgeneralization, etc (Beck, 1967, 1976; Beck and Weishaar, 2000). Empirically, cognitive distortions have been shown to be associated with depression, anxiety, and other affective symptoms (Leung and Poon, 2001; McGrath and Repetti, 2002; de Oliveira et al., 2015). Moreover, gender differences have been observed in various forms of psychological distress in which females were significantly over-represented (Hankin and Abramson, 2001; McLean et al., 2011).

Irwin and Bassham (2003) scrutinized ten cognitive distortions proposed by Beck (1967, 1976) and concluded that they each exhibit and epitomize at least one logical fallacy – an erroneous pattern of reasoning for arriving at the conclusion of an argument (Van Eemeren et al., 2009). The ability to identify and avoid making logical fallacy is indeed one of the key attributes of a critical thinker (Halpern, 2003). Although Irwin and Bassham (2003) made no claim that cognitive distortions are the results of weak reasoning ability, it raises the question of whether people with high critical thinking skills in general would experience fewer and less intense cognitive distortions and perhaps have better affective well-being. The point of particular interest here is whether critical thinkers can direct or generalize their critical thinking ability to evaluate their own thoughts and emotions.

### The Role Mindfulness Plays

The conceptual overlap of critical thinking and cognitive distortions has not been directly explored in the existing literature. One construct that is frequently associated with critical thinking and cognitive distortions, respectively, is metacognition. It is simply defined as “the monitoring and control of thought” (Martinez, 2006, p. 696). Kuhn (1999) views critical thinking as one of the sub-categories of metacognition known as “metacognitive knowledge” which can aid in the monitoring of thought. Scherer-Dickson (2004) postulates that cognitive distortions are both monitored and controlled by metacognition.

Recent research has highlighted the intricate relation between mindfulness and metacognition (Jankowski and Holas, 2014). Mindfulness is defined as one’s conscious awareness of the present moment in a non-judgmental manner (Kabat-Zinn, 1994). It is grounded in a perceptual, rather than cognitive or emotional manifestation of the current moment as it is. Shapiro et al. (2006) propose that mindfulness can bring about mental shifts in perspective which allow us to perceive our experience with enhanced clarity and objectivity. Such a shift in perspective is not only found to help regulate affects and behaviors but is also therapeutic in that it allows us to step back and observe our thoughts objectively without being controlled by them. Mindfulness can be measured as a trait or disposition (Brown and Ryan, 2003). Significant negative correlations have been demonstrated between trait mindfulness and depression (Cash and Whittingham, 2010), rumination (Raes and Williams, 2010), cognitive reactivity (Raes et al., 2009), social anxiety (Rasmussen and Pidgeon, 2011), and general psychological symptoms (Baer et al., 2006).

Cognitive distortions refer to both the frequency and intensity in which one engages in illogical thinking patterns (de Oliveira et al., 2015). As critical thinkers are good at evaluating arguments, making decisions, and solving problems, they should be less likely to commit thinking errors frequently, and intensely. However, this might not be the case for critical thinkers who are low in mindfulness. When people are too drowned in their thoughts and feelings (i.e., low in mindfulness), their metacognitive regulation ability is impaired (Jankowski and Holas, 2014), and they are more likely to commit cognitive distortions (Schütze et al., 2010). Critical thinkers are no exception. Even though critical thinkers know declaratively that cognitive distortions are illogical in theory, those with low mindfulness might still fall prey to committing cognitive distortions in everyday life due to their compromised metacognitive regulatory skills. Knowing and doing can be two different things. Furthermore, when critical thinkers with low mindfulness are unaware of their compromised critical thinking ability and continue to be self-assured in their judgment and decision, they are likely to believe in the cognitive distortions more strongly, which can then inflate their overall level of cognitive distortions.

### The Current Study

**FIGURE 1:**
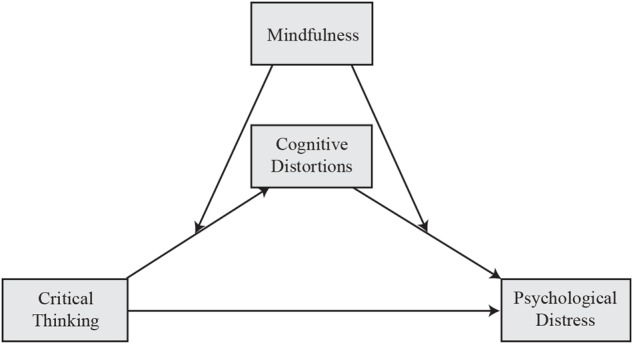
The proposed moderated mediation model.

The current study explored the interrelations between critical thinking, cognitive distortions, mindfulness, and psychological distress. Specifically, this study hypothesized that:

(1) Mindfulness moderates the relation between critical thinking and psychological distress. At low mindfulness, high critical thinking would predict high psychological distress, whereas the opposite direction of association would be expected at high mindfulness (i.e., negative relation between critical thinking and psychological distress).
(2) Cognitive distortions mediate the relation between critical thinking and psychological distress, moderated by mindfulness (see Figure 1). We postulate that at low mindfulness, critical thinking would more strongly predict cognitive distortions, which in turn predicts psychological distress. If the hypotheses of the present study hold true, high critical thinking ability could potentially be associated with psychological well-being when coupled with low mindfulness.

## Materials and Methods

### Participants and Procedures

The participants of this study consisted of 287 senior secondary school students (57% male and 43% female) from a local secondary school in Hong Kong. Ages ranged from 14 to 19 years, with a mean of 15.58 (*SD* = 0.85). Voluntary informed consent was obtained from the participants, their parents, and the school principal. Participants were guaranteed anonymity and confidentiality. Approval to conduct the study was granted by the research ethics committee at the university. Each participant attended a 40-minute session in group format in their respective classrooms to complete the measures during regular school hours. They were first allowed 15 min to fill out a questionnaire, which included demographic information (age, gender, and grade level), as well as measures of cognitive distortions, psychological distress, and mindfulness. They were then asked to complete the critical thinking assessment within a time limit of 25 min.

### Measures

#### Cognitive Distortions Questionnaire

Cognitive distortions were measured using the *Cognitive Distortions Questionnaire* (CD-Quest; de Oliveira et al., 2015). It is a 15-item six-point Likert scale ranging from “0” to “5” which assesses one’s overall level of cognitive distortions based on self-reported frequency and intensity of cognitive distortions committed during the past week. An example item on dichotomous thinking (also called all-or-nothing, black-and-white, or polarized thinking) reads: “I view a situation, a person or an event in “either-or” terms, fitting them into only two extreme categories instead of on a continuum.” The scoring for each item captured both the frequency of commission (from *No* to *Almost all of the time*) and the intensity of belief (from *A little* to *Very much*) (see Table 1). Higher scores indicate higher levels of cognitive distortions. The CD-Quest was translated by the first author into Chinese. A back-translation procedure was conducted to ensure the accuracy of the translation. Internal consistency reliability for the sample as measured by Cronbach’s alpha was 0.90.

**Table 1:** Example of the scoring format for cognitive distortions questionnaire (CD-Quest).

Frequency	No	Occasionally	Much of the time	Almost all of the time
**Intensity**				
I believe it…	0	–	–	–
A little	–	1	2	3
Much	–	2	3	4
Very much	–	3	4	5

#### Depression Anxiety Stress Scales

The 21-item version of the *Depression Anxiety Stress Scales* (DASS-21; Lovibond and Lovibond, 1995) was used to assess the psychological distress of the participants by measuring their level of depression, anxiety, and stress as three separate domains in the forms of cognitive, emotional, and physiological symptoms during the past week. Each domain consists of seven items. Self-report responses from the participants were scored on a four-point Likert scale (from “0” = *Never*, “1” = *Sometimes*, “2” = *Often*, to “3” = *Almost Always*), with higher scores indicating more anxiety, depression, and stress symptoms. Sample items include “I found it hard to wind down” and “I was aware of dryness of my mouth.” The Chinese version of the measure translated by Chan (2005) was used. Internal consistency reliabilities for the sample as measured by Cronbach’s alpha in the domains of depression, anxiety, and stress were 0.86, 0.76, and 0.81, respectively.

#### Mindful Attention Awareness Scale

The *Mindful Attention Awareness Scale* (MAAS; Brown and Ryan, 2003) was used to measure the participants’ trait mindfulness. It is a 15-item self-report Likert scale. Responses were scored on a six-point scale ranging from “1” (*Almost Always*), “2” (*Very Frequently*), “3” (*Somewhat Frequently*), “4” (*Somewhat Infrequently*), “5” (*Very Infrequently*) to “6” (*Almost Never*). Higher scores indicate higher mindfulness. A sample item reads “I find myself preoccupied with the future or the past.” The Chinese version of the MAAS translated by Zhang (2014) was used. Internal consistency reliability for the sample as measured by Cronbach’s alpha was 0.87.

#### Cornell Critical Thinking Test

A shortened version of the *Cornell Critical Thinking Test Level X* (CCTT; Ennis et al., 2005) was used to assess the critical thinking abilities of the participants. The test assessed four subscales of critical thinking: (1) Induction (Hypothesis Testing), (2) Credibility of Sources & Observation, (3) Deduction, and (4) Assumption Identification. Induction (Hypothesis Testing) refers to one’s ability to generalize from specific instances to form broad conceptual statements. Participants were asked in each item to judge if a fact supported a hypothesis, went against the hypothesis, or neither supported nor went against the hypothesis. Credibility of Sources & Observation measures the ability to decide whether or the extent to which one accepts an assertion without direct access to the basis of that assertion. In each of the items, participants were required to decide if one statement was more believable than the other or both were equally believable. Deduction is the ability to apply general rules to particular instances. Participants were asked to choose in each item the conclusion that followed necessarily from a given premise. Assumption identification is the ability to recognize assumptions which are the statements that fill the gaps in reasoning. Participants were asked in each item to identify an assumption which was taken for granted from a statement.

The original version of CCTT has 71 multiple-choice items and requires around 50 min to complete. Due to the time constraint imposed by the school for the data collection procedure, the use of the original version of CCTT was considered unfeasible. As a result, the CCTT was trimmed down to 33 items and the students were required to finish the items in 25 min. The full score for the test was 33 as each correct answer was scored one point. Five example questions with answers were provided to help the participants better understand the question format. Twenty-six undergraduate research interns (23% male and 77% female) were invited to complete the original 71-item CCTT in a pilot study (full score = 71). The internal consistency reliabilities of the overall CCTT score and those of the subscales of Induction, Credibility of Sources & Observation, Deduction, and Assumption Identification for the pilot sample as measured by Kuder–Richardson Formula 20 (KR-20) were 0.68, 0.63, 0.28, 0.54, and 0.31, respectively. The average score of the interns in the pilot was 48.08 (*SD* = 5.99, *Min* = 34, *Max* = 58). The interns’ quantitative performance and qualitative feedback were used to inform the difficulties of the items. The selection of the 33 items was guided by the original item-to-subscale ratio, the items’ level of difficulty, and resemblance to other items within the same subscale based on the professional judgment of the authors. Excluding example questions, the numbers of items in the subscales of Induction (Hypothesis Testing), Credibility of Sources & Observation, Deduction, and Assumption Identification were 10, 11, 7, and 5, respectively. The CCTT was translated by the first author into Chinese. A back-translation procedure was conducted to ensure the accuracy of the translation. Internal consistency reliability for the CCTT as measured by KR-20 was 0.58. The KR-20 for the subscales of Induction, Credibility of Sources & Observation, Deduction, and Assumption Identification were 0.30, 0.35, 0.30, and 0.21, respectively.

### Data Analysis

Structural equation modeling (SEM) was used to examine the hypothesized moderation and moderated mediation using IBM SPSS Amos version 25. Before testing the structural models, descriptive statistics and zero-order correlations of the study variables were computed. Proportions of missing data in the current study ranged from 0.1% (questionnaire data) to 0.8% (CCTT data). Little’s test indicated that the questionnaire data (χ^2^ = 939.53, df = 949, *p* = 0.58) and CCTT data (χ^2^ = 934.16, df = 953, *p* = 0.66) were all missing completely at random (MCAR). It is a statistical test that employs maximum likelihood estimation to assess if the missing values are related to any variables under study. A non-significant result on Little’s test indicates that there are no patterns in the missing data (i.e., MCAR). Mean imputation was used to replace the missing questionnaire data whereas missing data in the CCTT was scored as zero. SEM was used to test the proposed moderation and moderated mediation involving critical thinking and psychological distress as latent variables. Critical thinking was indicated by Induction, Credibility of Sources & Observation, Deduction, and Assumption Identification, whereas psychological distress was indicated by Depression, Anxiety, and Stress. Sum scores of the critical thinking subscales, cognitive distortions, mindfulness, and the subscales of psychological distress were entered as manifest variables. Model fit was assessed using chi-square test (χ^2^), comparative fit index (CFI), and incremental fit index (IFI), and root-mean squared error of approximation (RMSEA). For the cut-offs of the fit indices, CFI and IFI values larger than 0.95 (Bollen, 1989; Bentler, 1990) and RMSEA values less than 0.05 (Kline, 2004) are generally believed to indicate good model fit. To compare the moderation and moderated mediation models with the main effect models, the Akaike information criterion (AIC) and Browne-Cudeck Criterion (BCC) were also examined. Lower AIC and BCC values suggest better model fit. The significances of the direct and indirect effects were evaluated using bias-corrected bootstrap confidence intervals based on 1000 bootstrapping samples (Hayes, 2013). To further explore the moderating effect of mindfulness in the hypothesized models, the participants were categorized based on the MAAS z-scores into low mindfulness (1 *SD* below mean; *n* = 44), mid mindfulness (between -1 *SD* to +1 *SD*; *n* = 197), and high mindfulness (1 *SD* above mean; *n* = 47) subgroups. Multigroup moderation analyses (Byrne, 2016) were conducted using AMOS to examine differences in the strength of prediction under low and high mindfulness conditions. The interaction effects of the moderated mediation models were illustrated using the Johnson-Neyman plots, which help visualize the linear relationship between the independent and dependent variables across low and high mindfulness conditions.

## Results

### Descriptive Statistics

All raw data results are shown in Supplementary Table S1. The means, standard deviations, and the zero-order correlations among variables are shown in Table 2. Descriptive results showed that the overall CCTT score was modestly and positively correlated with anxiety (*r* = 0.12, *p* = 0.02) and stress (*r* = 0.10, *p* = 0.045). Cognitive distortions were found to be moderately and positively correlated with depression (*r* = 0.51, *p* < 0.001), anxiety (*r* = 0.54, *p* < 0.001), and stress (*r* = 0.63, *p* < 0.001) but negatively correlated with mindfulness (*r* = -0.54, *p* < 0.001). However, no significant correlations were observed between critical thinking and the measures of cognitive distortions, mindfulness, and depression. Gender was found to correlate significantly with anxiety (*r* = 0.17, *p* < 0.01) and stress (*r* = 0.15, *p* < 0.01). Age was found to correlate significantly with cognitive distortions (*r* = -0.13, *p* < 0.05). As such, gender and age were included as control variables in subsequent analyses.

**Table 2:** Descriptive statistics and zero-order correlations for the variables.

Variable	*M* (*SD*)	Possible score range	Reliability measure	1	2	3	4	5	6	7	8	9	10	11	12
1. CD-Quest	23.88 (14.25)	0–75	0.90 (α)	–											
2. CCTT	16.71 (4.13)	0–33	0.58 (KR-20)	0.08	–										
3. CCTT induction (hypothesis testing)	5.12 (1.73)	0–10	0.30 (KR-20)	0.04	0.65**	–									
4. CCTT credibility of sources & observation	5.94 (1.90)	0–11	0.35 (KR-20)	0.06	0.76**	0.29**	–								
5. CCTT deduction	3.46 (1.45)	0–7	0.30 (KR-20)	0.11*	0.63**	0.16**	0.31**	–							
6. CCTT assumption identification	2.19 (1.20)	0–5	0.21 (KR-20)	–0.03	0.54**	0.14**	0.22**	0.24**	–						
7. MAAS	59.50 (12.12)	6–90	0.87 (α)	–0.54**	–0.03	–0.05	–0.05	0.04	–0.00	–					
8. DASS-21 depression	5.37 (4.23)	0–21	0.86 (α)	0.51**	0.07	0.06	0.05	0.05	0.02	–0.47**	–				
9. DASS-21 anxiety	5.09 (3.55)	0–21	0.76 (α)	0.54**	0.12*	0.05	0.07	0.08	0.12*	–0.50**	0.72**	–			
10. DASS-21 stress	6.66 (4.10)	0–21	0.81 (α)	0.63**	0.10*	0.07	0.03	0.11*	0.06	–0.52**	0.72**	0.76**	–		
11. Age	15.58 (.85)	–	–	–0.13*	–0.09	–0.16**	–0.02	–0.07	0.04	–0.05	–0.03	–0.02	–0.06	–	
12. Gender	–	–	–	0.05	0.00	–0.04	–0.02	0.07	0.02	0.07	0.07	0.17**	0.15**	0.07	–

*CD-Quest, cognitive distortions questionnaire; CCTT, cornell critical thinking test; MAAS, mindful attention awareness scale; DASS, depression anxiety stress scales.^∗^*p* < 0.05; ^∗∗^*p* < 0.01.*

### Moderation Effect of Mindfulness on the Relation Between Critical Thinking and Psychological Distress

**FIGURE 2:**
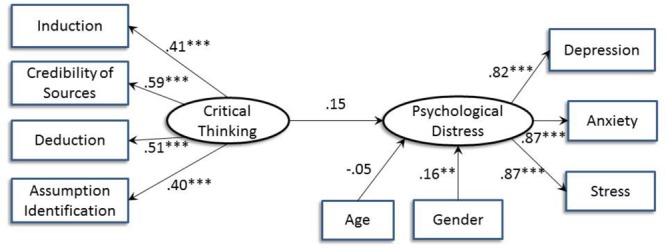
Structural equation model indicating the relation between critical thinking and psychological distress after controlling for age and gender. Standardized path coefficients are shown (^∗∗^*p* < 0.01; ^∗∗∗^*p* < 0.001).

Figure 2 shows the structural equation model on the relation between critical thinking and psychological distress with age and gender controlled for. The model was a good fit to the data with χ^2^ (26) = 32.73, *p* = 0.17; CFI = 0.99; IFI = 0.99; RMSEA = 0.03; AIC = 88.73; BCC = 90.76. The relation between critical thinking and psychological distress was not significant (*B* = 0.15, *p* = 0.08). The alternative model with psychological distress predicting critical thinking showed a slightly weaker model fit, with χ^2^ (26) = 37.99, *p* = 0.06; CFI = 0.98; IFI = 0.98; RMSEA = 0.04; AIC = 93.99; BCC = 96.01.

**FIGURE 3:**
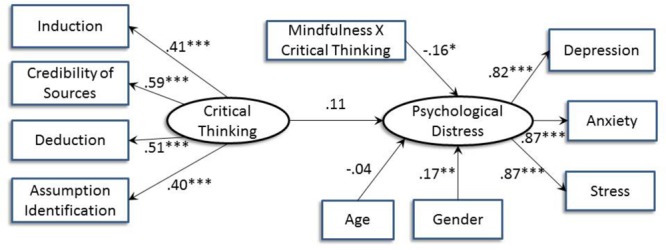
Structural equation model indicating the relation between critical thinking and psychological distress with mindfulness as the moderator after controlling for age and gender. Standardized path coefficients are shown (^∗^*p* < 0.05; ^∗∗^*p* < 0.01; ^∗∗∗^*p* < 0.001).

**FIGURE 4:**
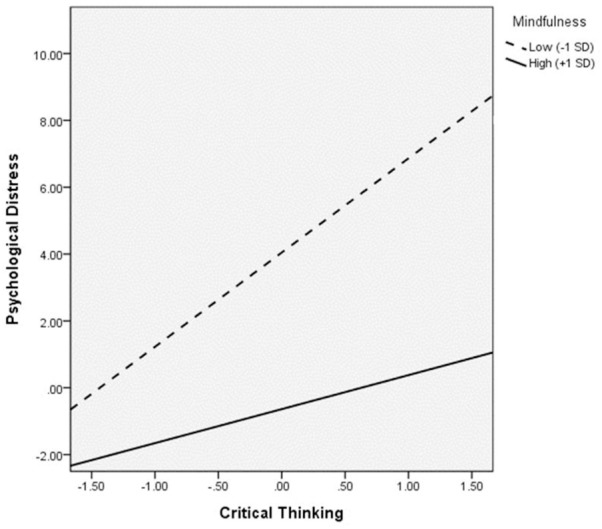
Johnson-Neyman plot showing the interaction effect of critical thinking and mindfulness on psychological distress.

Figure 3 shows the moderation effect of mindfulness. The interaction between mindfulness and critical thinking significantly predicted psychological distress (*B* = -0.16, *p* = 0.01), indicating a significant moderation effect of mindfulness on the relation between critical thinking and distress. Based on the multigroup analyses with mindfulness as the moderator, critical thinking was only significantly and positively associated with psychological distress when mindfulness was low (*B* = 0.51, *p* = 0.01). Figure 4 shows the interaction effect in which the positive relation between critical thinking and psychological distress was stronger for low mindfulness than high mindfulness.

### Moderation Effect of Mindfulness on the Relation Between Critical Thinking and Psychological Distress as Mediated by Cognitive Distortions

**FIGURE 5:**
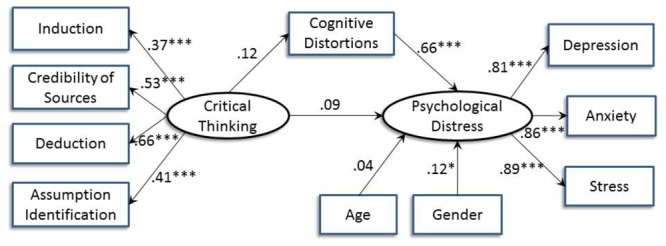
Structural equation model indicating the relation between critical thinking and psychological distress as mediated by cognitive distortions after controlling for age and gender. Standardized path coefficients are shown (^∗^*p* < 0.05; ^∗∗∗^*p* < 0.001).

The structural equation model on the relation between critical thinking and psychological distress as mediated by cognitive distortions with age and gender controlled for is shown in Figure 5. The mediation model was an acceptable fit to the data with χ^2^ (34) = 55.04, *p* = 0.01; CFI = 0.97; IFI = 0.97; RMSEA = 0.05; AIC = 117.04; BCC = 119.51. The indirect effect of critical thinking on psychological distress via cognitive distortions was non-significant [*B* = 0.08, BCCI (-0.02, 0.17)].

**FIGURE 6:**
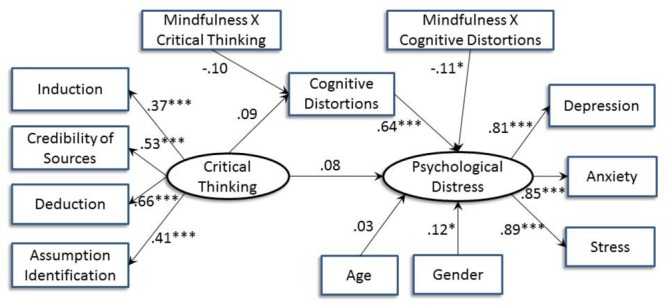
Structural equation model indicating the relation between critical thinking and psychological distress as mediated by cognitive distortions with mindfulness as the moderator after controlling for age and gender. Standardized path coefficients are shown (^∗^*p* < 0.05; ^∗∗∗^*p* < 0.001).

**FIGURE 7:**
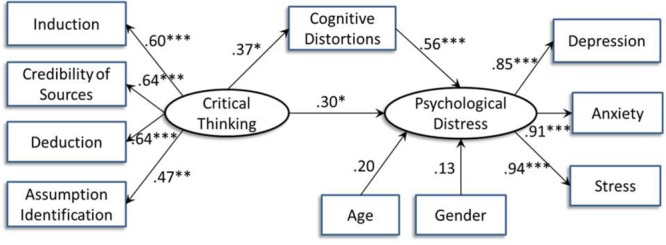
Structural Equation Model indicating the relation between critical thinking and psychological distress as mediated by cognitive distortions at low mindfulness condition after controlling for age and gender. Standardized path coefficients are shown (^∗^*p* < 0.05; ^∗∗^*p* < 0.01; ^∗∗∗^*p* < 0.001).

**FIGURE 8:**
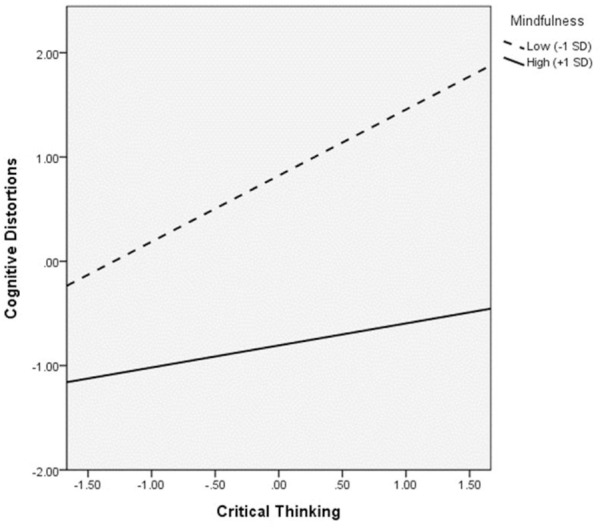
Johnson-Neyman plot showing the interaction effect of critical thinking and mindfulness on cognitive distortions.

**FIGURE 9:**
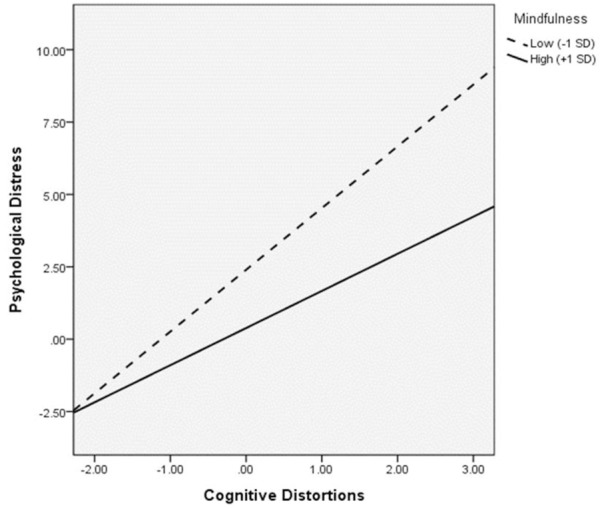
Johnson-Neyman plot showing the interaction effect of cognitive distortions and mindfulness on psychological distress.

On testing the moderation effect of mindfulness (Figure 6), interaction of mindfulness and cognitive distortions significantly predicted psychological distress (*B* = -0.11, *p* = 0.02), whereas mindfulness X critical thinking marginally predicted cognitive distortions (*B* = -0.10, *p* = 0.08). The indirect effect of critical thinking on psychological distress via cognitive distortions was only significant at low mindfulness [*B* = 0.21, BCCI (0.09, 0.41)]. Figure 7 shows the moderated effect of mindfulness on the relation between critical thinking and psychological distress as mediated by cognitive distortions in low mindfulness condition. At low mindfulness, increases in critical thinking were significantly associated with increases in cognitive distortions, which in turn predicted higher psychological distress. As the direct effect of critical thinking on psychological distress [*B* = 0.30, BCCI (0.004, 0.54)] was significant, incomplete mediation via cognitive distortions took place. Johnson-Neyman plots demonstrating the cross-over interaction effect of critical thinking and mindfulness on cognitive distortions and that of cognitive distortions and mindfulness on psychological distress are shown in Figure 8, 9.

## Discussion

The aim of the current study is to explore the interrelations between critical thinking, cognitive distortions, mindfulness, and psychological distress. We hypothesized that there existed a moderated mediation among the variables. Results from the current study supported Hypothesis 1 that mindfulness moderated the relation between critical thinking and measures of psychological distress. The findings relating to Hypothesis 2 showed that the relation between the study variables was more nuanced than the original hypothesis. In low mindfulness conditions, critical thinking was positively associated with cognitive distortions which could in turn give rise to psychological distress in the form of anxiety, depression, and stress. The moderating roles of mindfulness tested in the hypotheses were clearly demonstrated.

What is of particular interest is one possible interpretation of the findings. Critical thinkers are believed to have good metacognitive knowledge (Kuhn, 1999). However, metacognitive knowledge is insufficient in itself in reducing cognitive distortions without effective metacognitive regulation. Mindfulness brings about mental shifts in perspective (Shapiro et al., 2006) which is thought to enhance metacognitive regulation (Wells, 2000). As such, though critical thinkers have good metacognitive knowledge, but when coupled with low mindfulness, they might still fall victim to cognitive distortions.

As suggested in the introduction, the reason why critical thinkers who are low in mindfulness might experience more overall cognitive distortions is because they are unaware that their good critical thinking ability only serves them well in monitoring but not in regulating their cognition. When critical thinkers fall into thinking traps, they might still continue to be self-assured in their judgment and believe in the cognitive distortions even more strongly, as their self-efficacy on critical thinking is high due to their previous successful experiences. Since the construct of cognitive distortions consists of both a frequency and an intensity component, increase in either component can inflate the overall level of cognitive distortions.

Both Hypotheses 1 and 2 predicted that at high mindfulness, the relation between critical thinking and psychological distress would be negative. As mentioned earlier, mindfulness was hypothesized to allow a critical thinker to step back from his or her own consciousness and view accurately his or her thoughts from a third person’s perspective. However, our results revealed that the predicted negative relation between critical thinking and psychological distress at high mindfulness was not significant. We propose here three plausible explanations for the non-significant results observed which are related to the properties of the sample, the overconfidence and self-assuredness of critical thinkers, and the complexity of cognitive distortions.

First of all, the overall level of trait mindfulness among this sample of adolescents might not be sufficiently high for the effect of mindfulness to become apparent in the said relation. As seen from the moderation results, the positive associations of critical thinking with psychological distress indeed weakened with increases in mindfulness. Similar decreases in the strength of correlations were also observed in the moderated mediation models. The Johnson-Neyman plot (Figure 8) shows that the positive relation between critical thinking and cognitive distortions declines with increases in mindfulness. It is possible that when the trait mindfulness level of the overall sample is high enough, the relation between critical thinking and psychological distress could be significantly negative. However, as our sample was not representative of the population, we were unable to determine if the trait mindfulness measure of the sample is at a level comparable with that of the population. Trait mindfulness has been shown to increase with age (Shook et al., 2017). Future studies may test the mindfulness-moderated effect of critical thinking on psychological well-being among older age groups using representative samples to further examine this postulation.

Second, the possible adverse effect of critical thinking on promoting cognitive distortions – which may be related to critical thinkers’ overconfidence and self-assuredness – might have overweighed its beneficial effect in alleviating cognitive distortions through the identification of logical fallacies (Irwin and Bassham, 2003). Exercising critical thinking to correct logical fallacies could to some extent prevent cognitive distortions by enhancing metacognitive knowledge. However, its effect might be overshadowed by the plausible problem relating to critical thinkers’ self-assuredness, which might further undermine metacognitive regulation in low mindfulness conditions. If mindfulness is low, critical thinkers may not be aware that their metacognitive knowledge is insufficient to contribute to effective metacognitive regulation, but rather continue to be self-assured in their critical thinking due to their previous successful experiences.

Third, cognitive distortions do not exclusively represent logical fallacies. Having high critical thinking and high trait mindfulness might allow one to recognize and rectify the illogicality of cognitive distortions, but their underlying assumptions and beliefs might not be addressed. According to Beck (2011), cognitive distortions are the manifestation of people’s rigid and unrealistic appraisal of themselves and others. If this is indeed the case, tackling logical fallacies *per se* might not suffice to eliminate cognitive distortions. For instance, the cognitive distortion called dichotomous thinking exemplifies the logical fallacy of false dilemma which presumes that only two alternatives exist when there are in fact more. However, the underlying assumption giving rise to dichotomous thinking might be due to a way of evaluating oneself or others in either all good or all bad terms with nothing in between. In this case, only resolving the logical fallacy might not be enough to challenge one’s unrealistic appraisal of oneself or others.

### Limitations and Future Directions

The current study has several limitations that are worth noting. First, the internal consistency of the critical thinking measures used in the study was low, with a KR-20 of 0.58. The findings in this study should be interpreted with caution. The low internal consistency might be due to the trimming down of the original 71-item scale into a 33-item one. Test performances of undergraduate research interns in the pilot study were used to inform the trimming down of the test items. Preferably, secondary school students should have been assessed instead. Moreover, the scale had not been validated in the local Hong Kong population. Further studies should adopt critical thinking scales with better reliability and validity to assess critical thinking. However, we wish to highlight that according to the test manual of the CCTT, Ennis et al. (2005) point out that critical thinking tests in general have lower internal consistencies than other constructs due to the heterogeneity of critical thinking. Verburgh et al. (2013) demonstrated in a validation study of the CCTT and Halpern critical thinking assessment (HCTA) that the internal consistencies of the full CCTT and HCTA were as low as 0.52 and 0.49 and that of the subscales of CCTT and HCTA were as low as 0.30 and 0.34, respectively.

Furthermore, the study of Scott (1983) was inconclusive in showing that anxiety might be the independent variable giving rise to impaired critical thinking. We are unable to rule out this alternative explanation due to the correlational nature of our study. Nevertheless, our results showed that the increases in psychological distress, as opposed to Scott’s supposition, was associated with higher critical thinking performance. A possible explanation could be that a certain degree of psychological distress serves as a catalyst for self-reflection, keeps the brain alert (Kirby et al., 2013), and boosts cognitive performance. The causal relations among critical thinking, cognitive distortions, and psychological distress await further investigation using experimental designs through critical thinking training.

Finally, although the definition of critical thinking makes it clear that it is different from “criticism” or “fault-finding,” there might exist a certain degree of overlap. After all, a good critical thinker is likely to be able to detect more faults in oneself or others than his or her less critical counterparts. The relations between critical thinking, self-criticism, and criticism toward others can be further explored.

### Conclusion and Implications

Critical thinking is a highly sought after 21st century skill that allows people to make good judgment and decisions. Its benefits on people’s cognitive functioning have been well-documented. However, less is known about its impact on people’s affective functioning. This study revealed that high critical thinking skills were associated with high psychological distress in low mindfulness conditions. Cognitive distortions were shown to be the path through which critical thinking was associated with psychological distress in low mindfulness conditions. When trait mindfulness was low, high critical thinking was significantly associated with high cognitive distortions and high psychological distress.

The findings of the current study are impactful as they suggest that in low states of mindfulness, critical thinking might lead to cognitive distortions which in turn give rise to psychological distress. From an educational point of view, critical thinking skills are indispensable skills to be taught to students. The key here is to maximize the benefits of critical thinking on cognitive functioning while minimizing the negative associations critical thinking has on psychological well-being. From the results of this study, it can be seen that mindfulness might benefit critical thinkers in terms of their psychological well-being. Recent research has shown that trait mindfulness can be enhanced through mindfulness-based intervention such as the b (“Dot be”) Curriculum developed by the Mindfulness in Schools Project (Kuyken et al., 2013). We therefore wish to argue that mindfulness-based training should perhaps become an integral part of students’ development of social-emotional competency in schools in order to enhance their overall psychological well-being.

## Ethics Statement

All subjects gave written informed consent in accordance with the Declaration of Helsinki. The protocol was approved by the institutional research committee of The University of Hong Kong.

## Author Contributions

MS conceived the idea, carried out the implementation, and wrote the manuscript. KS supervised the project and helped in analyzing the data and writing the manuscript.

## Conflict of Interest Statement

The authors declare that the research was conducted in the absence of any commercial or financial relationships that could be construed as a potential conflict of interest.

## Supplementary Material

TABLE S1 Raw data results of the study.
